# Design of a Concrete Shear Device and Investigation of the Shear Performance of New-to-Old Concrete Interfaces

**DOI:** 10.3390/ma18174164

**Published:** 2025-09-05

**Authors:** Jianglei Tian, Ruyu Li, Tonghao Wu, Min Zhang, Yangyang Xia, Jizhi Huang

**Affiliations:** 1 College of Water Conservancy and Civil Engineering, South China Agricultural University, Guangzhou 510642, China; zhoujingwen@mail.gjtjt.com (J.T.); 20233181008@stu.scau.edu.cn (R.L.); 13620185246@163.com (T.W.); ammindy@scau.edu.cn (M.Z.); 2 Guangzhou Expressway Co., Ltd., Guangzhou 510320, China; xiayangyang@mail.gjtjt.com

**Keywords:** concrete reinforcement, shear test, surface roughness, finite element simulation, interfacial bond performance

## Abstract

Shear strength, which indicates the interfacial bond performance between new and old concrete, is critical in the field of structural reinforcement and rehabilitation. However, the absence of standardized testing equipment has hindered the accurate quantification of this parameter. To address this gap, a dedicated shear-loading apparatus was designed in this study, and finite element modeling was conducted to simulate the shear performance of concrete with different interface roughness. The results show that failure consistently occurs at the interface and that roughness has significant influence on shear capacity. In order to reveal the relationship between shear strength and surface roughness, shear experiments were conducted on new–old concrete using the device we designed. The surface of old concrete was treated by water-jetting, electric hammering, grooving, or grout seal strip to create different profiles, the roughness was quantified by 3D scanning and Fourier transform analysis, and fresh concrete was then cast atop the processed surfaces to form composite specimens. The results show that the correlation between shear strength (τ) and Fourier transform roughness (FTR) can be described with the equation τ (MPa) = 0.546*FTR*^2^ + 1.832*FTR* − 0.447.

## 1. Introduction

Concrete, consisting of cement, sand and gravel, is one of the most important building materials in the world. Compared to other materials, concrete exhibits excellent mechanical performance and durability in different environmental conditions. However, under the effect of loading and environmental erosion, aging and deterioration are inevitable, so surface repair or structural strengthening become necessary for concrete structures after a certain period of service. Currently, casting new concrete onto the surface of processed old concrete is the most widely accepted fixing approach. In most protective restoration conditions, adhesive strength is the most important factor for the patching material to avoid peeling off [1,2,3]. However, for other reinforcement conditions, such as piers and columns, the structures are perpendicular to ground and may subjected to shear forces, so they need not only adequate bond strength but also high shear strength. Currently, the methodology to test bond strength has been well-established, and reliable results can be obtained by tensile tests [4,5,6,7] and splitting tests [8,9,10,11,12]. However, methods for testing shear strength are relatively limited, standardized equipment has not been proposed, and non-standard equipment exhibits significant shortcomings. So, there is an urgent need to develop a reliable and efficient device for testing shear strength.

**Figure 1:**
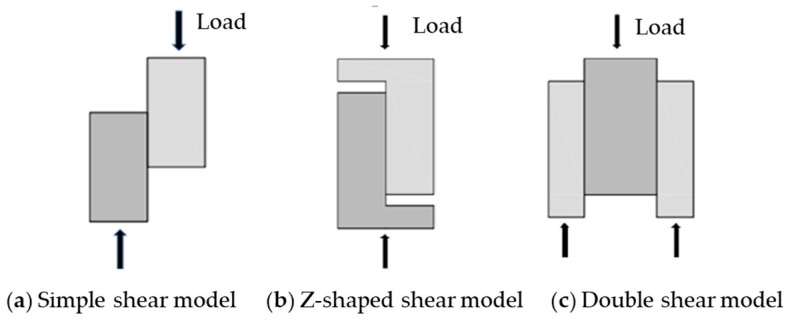
Traditional methods in concrete interface shear testing.

Traditional approaches to measure interfacial shear strength include the direct shear test [13,14,15] and skew shear test [16,17,18]; the direct shear test is further categorized into the simple shear test and double shear test. The principle of original simple shear test is illustrated in Figure 1a. A pair of shear forces is applied to the opposite sides of the specimen, and shear strength can be obtained by measuring the failure load. For this approach, the asymmetric loading configuration is prone to inducing additional bending moments, leading to stress concentration and significant variability in testing results. To address this problem, Hofbeck et al. [19] proposed a Z-shaped shear specimen in 1969, as shown in Figure 1b. Compared to the original simple shear test model, this Z-shaped specimen can reduce the bending stresses caused by eccentric loads. Although the Z-shaped model is an improvement over the simple shear test, the fabrication of the specimen is more complicated and dimensional deviation also leads to inaccurate results. Moreover, additional devices are also needed to provide lateral pressure to minimize the effect of bending stress in the Z-shaped model. Compared to the simple shear test, the double shear test [20,21] (shown in Figure 1c) is less affected by bending stress and yields more reliable results. The double shear specimen features a symmetrical configuration with two shear planes where the bending moments generated on two interfaces counteract each other during loading. However, the fabrication of double shear specimens is also complicated and the specimen often randomly fails at only one side.

In order to address the shortcomings of traditional equipment and improve the accuracy and efficiency of the shear strength test, this study designed a novel testing apparatus. The feasibility and reliability of the device were validated through finite element modeling and experimental testing. Surface roughness is reported to be the most critical parameter governing interfacial bond strength among various factors such as surface wetness, concrete age, etc. To quantitatively characterize the relationship between surface roughness and shear strength, different methods were employed to create varying levels of roughness on old concrete samples, and new concrete was cast on the processed surface to create composite old–new concrete shear specimens [22,23,24,25]. Using the developed apparatus, this study systematically examined the relationship between interface roughness and shear strength.

## 2. Materials and Methods

### 2.1. Design of Shear Testing Device and Testing Method

#### 2.1.1. Device Design

**Figure 2:**
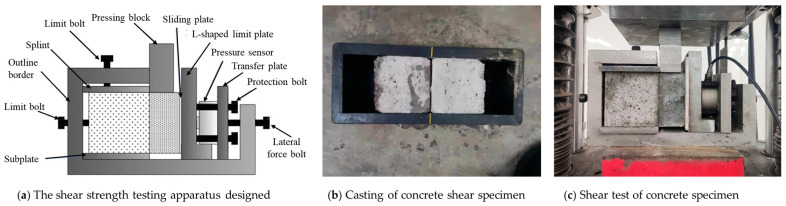
The shear strength testing apparatus designed in this study and testing method. (**a**) Details of the shear strength testing apparatus designed. (**b**) Casting of concrete shear specimen in a standardized mold. (**c**) Shear test of a concrete specimen using the apparatus designed in this work.

Considering the characteristics of the concrete in shear test, the device should meet the following requirements. Firstly, limiting the position of the shear specimen during the test. Secondly, providing a controllable lateral force and minimizing the friction between the specimen and the device. Based on the above points, the shear testing device designed in this study is shown in Figure 2a.

The device primarily consists of a metal fixture and a lateral force system. The metal fixture is designed to provide constrained support for specimens, it comprises a steel frame, clamping plates, and limit bolts. The steel frame, fabricated from 25 mm thick No. 45 steel, can prevent deformation during loading. The clamping plates, i.e., the splint and subplate in Figure 2a, along with the limit bolts, help to limit the position of the specimen and ensure it fails at the designated interface. The lateral force system primarily consists of a lateral force bolt, a transfer plate, and a pressure sensor. When the bolt is tightened, lateral force will be transferred through the transfer plate, pressure sensor, and L-shaped plate to the concrete specimen. With the help of pressure sensor, the lateral force can be precisely controlled by the lateral force bolt to ensure all specimens are testing under a consistent lateral force. In order to minimize the friction between the specimen and the L-shaped limit plate, a polytetrafluoroethylene (PTFE) plate is placed on the right side of the specimen. The friction coefficient between PTFE and steel typically ranges from 0.2 to 0.3, and the application of lubricant can further reduce the friction coefficient.

#### 2.1.2. Fabrication and Casting of Shear Specimens

As shown in Figure 2b, the shear specimens with new-to-old concrete interfaces were prepared using laboratory molds according to the following procedure:

(1) Surface processing of old concrete: The old concrete, which was initially cast using a 100 mm × 100 mm × 100 mm mold, had its surface roughened using different methods to achieve the desired surface texture.
(2) Casting new concrete: Two old concrete specimens were placed in the middle of a 300 mm × 100 mm × 100 mm mold with their roughened surfaces facing outward. Fresh concrete was then cast into the end of the mold to form a new-to-old concrete composite specimen. After demolding, the specimens were cured under standard conditions. Using this method, two shear specimens can be made simultaneously in a single mold. Note that the old concrete must be positioned centrally to ensure equal volume distribution between the two shear specimens.

#### 2.1.3. Testing Method for Shear Specimens

As shown in Figure 2c, the assembly and testing process of the shear apparatus was conducted as follows:

(1) Installation of specimen: The cured concrete specimen was positioned in the device between the splint and subplate, the interface was aligned with the edge of the subplate to guarantee that the failure plane is right on the interface. The limit bolts were then tightened to fix the specimen.
(2) Installation of sliding plate: A PTFE plate with grease coated on the right side was set between the concrete and the L-shaped steel plate to reduce the fiction between the two materials.
(3) Installation of pressure sensor and protection device: The pressure sensor was mounted on the L-shaped plate. The pressure is controlled by the lateral force bolt. The protection bolts were adjusted to maintain proper clearance from the L-shaped plate, thus preventing the pressure sensor from overloading and potential damage.
(4) Shear test: After assembly, the device was installed on the Mechanical Testing and Simulation (MTS) machine. A vertical load with a constant loading rate of 0.2 kN/s was applied through the pressing block, and the failure load and load–displacement curve were recorded by the MTS.

The shear strength of the new–old concrete interface is calculated using Formula (1):

(1) $$\tau =\frac{P_{u}}{A}$$

where *τ* is the interfacial shear strength, in MPa; *P_u_* is the ultimate load value, in N; and *A* is the area of the new–old concrete interface, in mm^2^.

### 2.2. Composition and Mechanical Properties of New and Old Concrete

In this work, the old concrete to be repaired was simulated using laboratory-made concrete with a strength grade of C40 and a size of 100 mm × 100 mm × 100 mm. The cement used in the old concrete is a commercial product of P.O 42.5 cement from Shijing Ltd., Guangzhou, China. The sand is a medium sand from Beijiang River in Guangzhou, with a fineness modulus of 2.8, and the gravel is granite stone with diameters ranging from 5 mm to 30 mm. The composition of the old concrete and its mechanical properties are shown in Table 1. It should be noted that the shear test was conducted when the concrete was 60 days old, so the compressive strength was 54.6 MPa.

As for the new concrete, it is stipulated in Chinese standards that the strength of repair materials should be higher than the substrate concrete by one grade. In engineering practice, mortar is more widely accepted than concrete, since mortar has better homogeneity and higher bond strength with substrate concrete. In this work, a commercial dry-mixed mortar produced by Beijing Sino-sina Building Technology Co., Ltd. (Beijing, China) was employed as the repair material to form old–new composite concrete shear specimens. The shear test was conducted 7 days after casting onto old concrete surface, and the 7d compressive strength of newly cast mortar is 65.12 MPa.

### 2.3. Concrete Surface Roughening Treatment and Characterization of Roughness

#### 2.3.1. Surface Roughening Treatment

**Figure 3:**
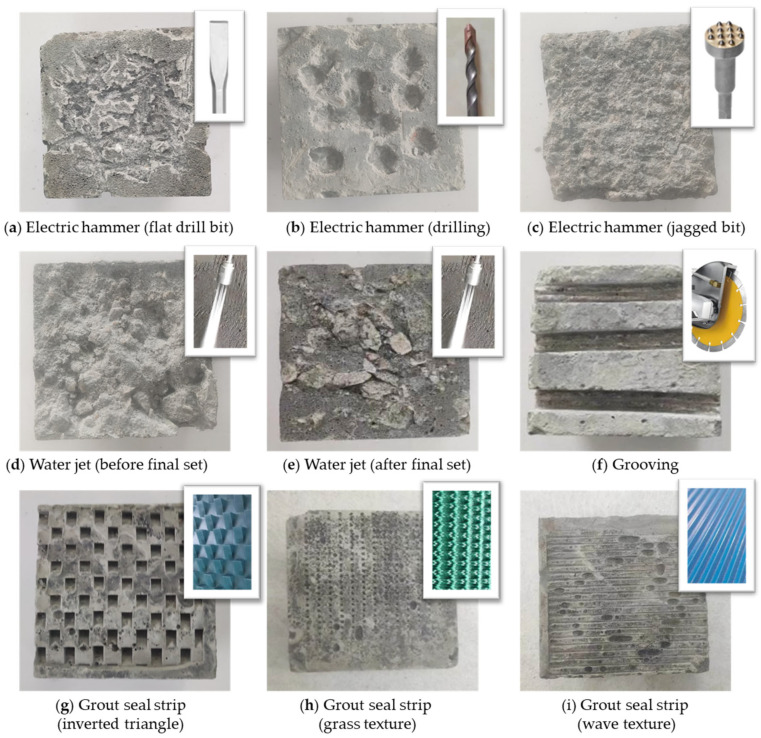
Concrete surface morphologies obtained by different treatment methods.

In this paper, electric hammering, water-jetting, grooving, and grout seal strip were used to roughen the surfaces of old concrete. The morphologies of concrete surfaces obtained using the different treatment methods are shown in Figure 3.

#### 2.3.2. Characterization of Concrete Surface Roughness

**Figure 4:**
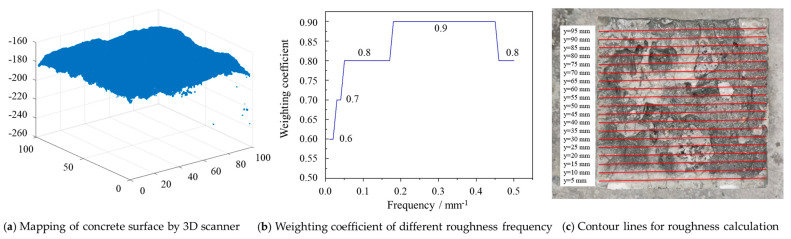
Characterization of concrete surface roughness. (**a**) The 3D coordinate surface of concrete obtained by HSCAN PRINCE775 scanner. (**b**) Weighting coefficient of different roughness frequencies. (**c**) Contour lines selected for roughness calculation.

The treated concrete surfaces were scanned using the HSCAN PRINCE775 handheld 3D scanner with a scanning precision of 0.2 mm. Figure 4a exhibits a typical 3D coordinate surface for concrete obtained by the scanner.

Based on the 3D coordinate data for the concrete surface collected above, this paper uses the contour line roughness algorithm from existing research to calculate the roughness [26]. The calculation formula is as follows:

(2) $$FTR=\sum_{j=1}^{m}(g_{j}AM_{j})$$

where *FTR* is the abbreviation of Fourier transform roughness, *AM_j_* is the amplitude of the *j* frequency, and *g_j_* is the weighting coefficient corresponding to the *j* frequency, as shown in Figure 4b. *j* = 1, 2, 3, …m (m is the number of identified frequencies).

The *FTR* calculated from Formular (2) is the roughness of a single contour line. In order to obtain the roughness of a concrete surface, 19 contour lines were extracted from the 3D coordinate data at an interval of 5 mm, as shown in Figure 4c. Fourier transforms were performed on each contour line to obtain the frequency domain graph for each contour line (i.e., Figure 4b), then the *FTR* of each contour line can be obtained. The mean *FTR* value of all 19 contour lines on each surface is the roughness of the concrete surface.

### 2.4. Finite Element Simulation

#### 2.4.1. Material Definition

A finite element simulation analysis of the aforementioned loading model was conducted using ABAQUS 2019. The concrete damaged plasticity (CDP) model was adopted to describe the concrete constitutive relation. The new and old concretes were defined with different characters in finite element simulation. The old concrete on left side is normal concrete (NC) with a density of 2.4 g/cm^3^, an elastic modulus of 3.60 × 10^3^ MPa, a Poisson’s ratio of 0.2, and a peak compressive strength of 40 MPa. The new concrete on the right side is ultra-high-performance concrete (UHPC) with a density of 2.4 g/cm^3^, an elastic modulus of 4.05 × 10^3^ MPa, a Poisson’s ratio of 0.2, and a peak compressive strength of 50 MPa.

The CDP model also requires the definition of the following plastic parameters: the dilation angle *ψ*, flow potential eccentricity *ε*, strength ratio under uniaxial and biaxial states *f_b_*_0_*/f_c_*_0_, invariant stress ratio *κ*, and viscosity coefficient *μ*. Herein, the *ψ* and *ε* control the shape of the non-associated flow potential function, while *f_b_*_0_*/f_c_*_0_ and *κ* control the yield surface of concrete. The *μ* is a viscoplastic regularization parameter used to improve the convergence difficulties of the model due to the softening and stiffness degradation behavior of materials. The specific values of each parameter are shown in Table 2 [27].

#### 2.4.2. Definition of Interface Properties

**Figure 5:**
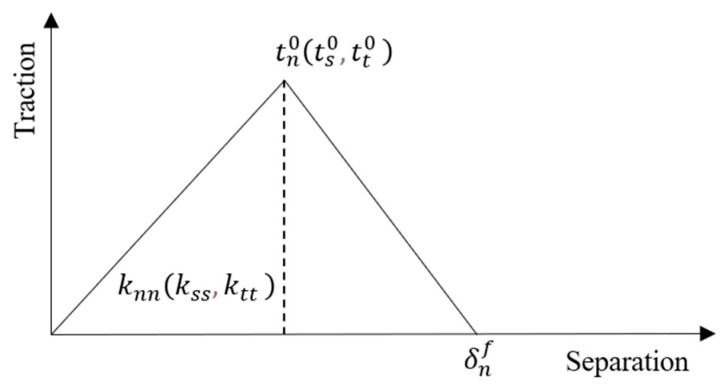
Cohesive force model applied in defining interface properties.

The constitutive model of the interface connection used in this paper is the traction-separation model, which is a cohesive model assuming the interface has zero thickness. It is assumed that when the interface satisfies the damage criterion of Equation (3), damage begins to occur. After damage occurs, the damage assessment is determined based on the total fracture energy or the total plastic displacement at failure, as shown in Figure 5.

(3) $${\left(\frac{t_{n}}{t_{n}^{0}}\right)}^{2}+{\left(\frac{t_{s}}{t_{s}^{0}}\right)}^{2}+{\left(\frac{t_{t}}{t_{t}^{0}}\right)}^{2}=1$$

In the equation, *t_n_*, *t_s_*, and *t_t_* represent the maximum values of contact stress in three directions. *t_n_* is the contact stress in the normal direction at the interface, *t_s_* is the shear contact stress in the first shear direction, and *t_t_* is the shear contact stress in the second shear direction.

The existing literature has calibrated the finite element parameters of the interface under three roughness levels, and it is believed that the normal and tangential stiffness are the same under the three roughness levels. However, considering that the normal stiffness should be different under different roughness levels, adjustments are made based on the following formula [28]:

(4) $$K_{nn}=\frac{P}{A\delta },$$

In the equation, *P* represents the applied load, *A* represents the bonding interface area, and *δ* represents the displacement that occurs at the bonding interface.

The relationship between tangential stiffness parameters *K_ss_* and *K_tt_* and normal stiffness *K_nn_* is given by

(5) $$K_{ss}=K_{tt}=\frac{K_{nn}}{2(1+v)},$$

In the equation, *ν* is the Poisson’s ratio, which is taken as 0.17 here. *K_nn_*, *K_ss_*, and *K_tt_* are the stiffness components related to the normal direction and shear directions through the interface before damage begins.

When calculating the tangential stiffness using the above equation, the UHPC-NC interface is considered a uniform thin layer and the tangential stiffness is affected by the normal stiffness, which facilitates the determination of the tangential stiffness value.

In order to reveal the correlation between surface roughness and shear strength, the roughness of different interfaces was characterized with different parameters that are referenced from the research of Li [16]. The interface property parameters for three different roughness levels are shown in Table 3.

#### 2.4.3. Definition of Boundary Conditions

**Figure 6:**
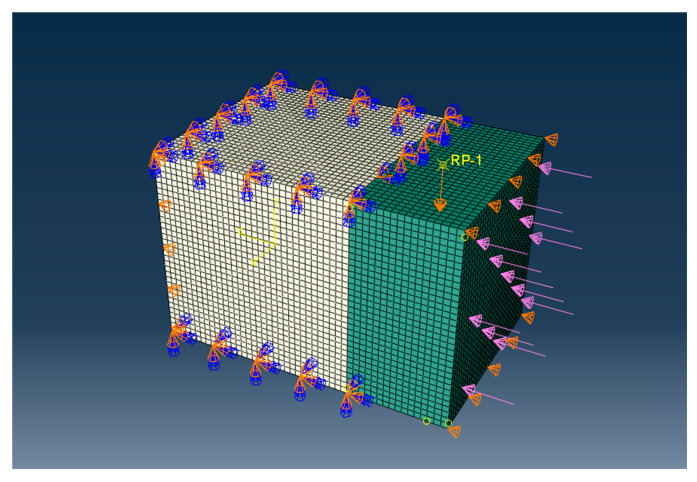
Finite element model and boundary conditions.

The boundary conditions were established in accordance with the actual constraints and loading conditions in shearing test, as depicted in Figure 6. The top and bottom surfaces of NC were rigidly fixed, and the left side was constrained to restrict transverse displacement. A lateral constraint was applied to the right side of the UHPC to prevent lateral translation or tilting, and a small transverse load of 100 N was also applied on the right side of the UHPC to ensure close contact between the UHPC and the PTFE plate during testing. This load can also minimize the friction during the test. Given that the coefficient of friction between the PTFE plate and steel is less than 1, the friction during the shear test is below 100 N, which is much smaller than the failure load in the shear test (less than 0.5%) and has negligible influence on the testing result.

The shear load was applied through displacement control at the loading point on top of the UHPC in the vertical direction, with a displacement magnitude of 2 mm. This displacement value was selected to ensure failure at the interface and clear observation of the failure zones in the strain contour plots. The loading process was divided into 10,000 incremental steps for an iterative solution, with minimum and maximum increment sizes of 0.00005 mm and 0.5 mm, respectively. The computation was terminated when the displacement reached 2 mm.

## 3. Results and Discussion

### 3.1. Finite Element Simulation Results

#### 3.1.1. Stress Nephogram

**Figure 7:**
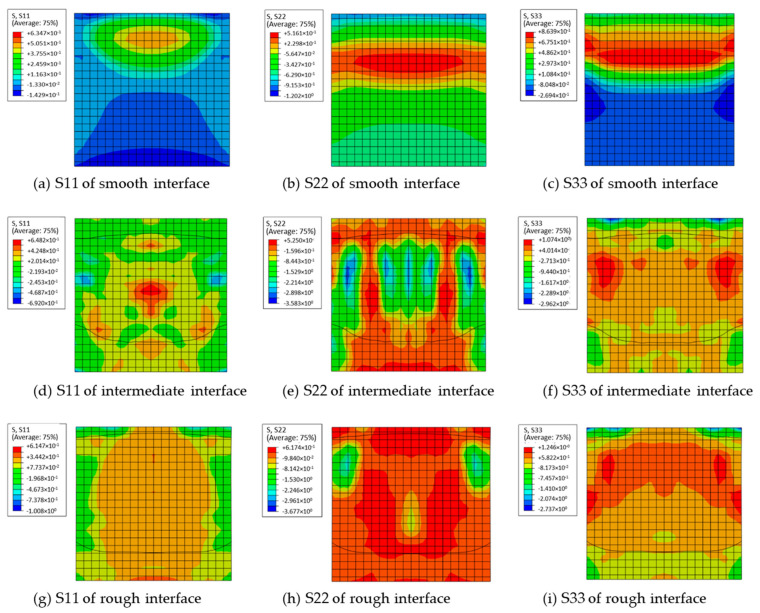
Stress nephogram of concrete interfaces with different roughnesses.

The stress distributions of concrete models with different roughnesses are shown in Figure 7, and the maximum normal stresses in different directions are presented in Table 4. Herein, the S11, S22 and S33 represent the normal stress in the horizontal direction, vertical direction and the direction perpendicular to the interface at the UHPC-NC interface, respectively.

We can see from Figure 7 that, surface roughness has significant influence on the stress distribution of concrete in shearing test. With the increase of surface roughness, the region under tensile stress extends gradually from the upper to the lower part. Obviously, the increasing roughness enhanced the bonding strength between new and old concrete, as a result, more area on interface will be subjected to tensile stress during shear test. We can also see from Table 4 that, the maximum compressive stress of S11, S22 and S33 increases continuously with increasing roughness. This is because, the concrete model in this work bears both shear and bending force due to eccentric loading. The increasing roughness improved the bonding strength on interface and resulted in a higher failure load, which leaded to a higher bending force and increased the compressive stress on compression zone. Similar result can be observed from the results of tensile stresses S22 and S33, which also result from the improved failure load and bending force.

#### 3.1.2. Strain Nephogram

**Figure 8:**
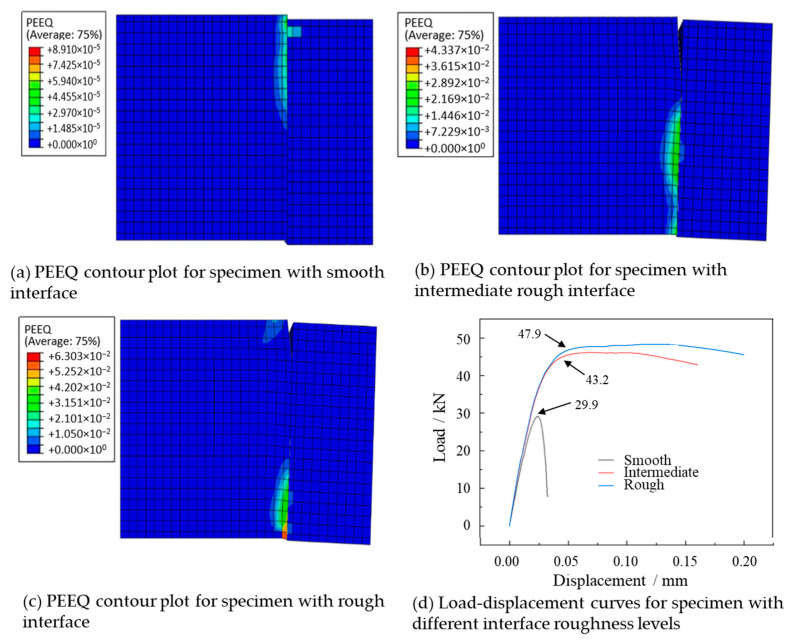
PEEQ contour plots of concrete samples with different roughness levels and their load-displacement curves.

Plastic Equivalent Strain (PEEQ) is a key indicator in ABAQUS 2019 for evaluating the plastic state of materials [29,30]. The value greater than zero indicates the onset of element yielding, while the maximum value signifies that the material is approaching failure. As we can see from the PEEQ contour plots in Figure 8, the maximum values of PEEQ all occur at the interface and are biased towards the NC side regardless of surface roughness. This is because the UHPC has higher strength and toughness, which can resist the extension of the failure region towards the UHPC side. Furthermore, we can also see from Figure 8 that with the increase of roughness, the location of preferential failure gradually shifts from the upper part to the lower part of the interface due to the dilatancy effect of concrete.

The load–displacement curves for UHPC-NC shear specimens with three different roughness levels are shown in Figure 8d. The failure loads of the specimen with smooth, intermediate and rough interface are 29.9 kN, 43.2 kN and 47.9 kN, respectively, indicating that the increasing roughness can improve the shear capacity of new-to-old concrete interface.

### 3.2. Interface Failure Mode

In our experimental test, the surface of old concrete was treated by different approaches, the treatment method can change not only the surface roughness but also the failure mode on interface. In this work, the surface roughness was characterized by FTR, and the failure mode was classified based on the characteristics of failure morphology, which can be divided into the following three modes:

Mode A: Interfacial failure. In this mode, the concrete primarily fails at the interface, and there is little concrete adheres to each other;

Mode B: UHPC failure. There is a lot of UHPC adheres to the surface of NC;

Mode C: NC failure. There is a lot of NC adheres to the surface of UHPC.

The average FTRs of concrete specimens treated by different methods and their failure modes are shown in Table 5.

**Figure 9:**
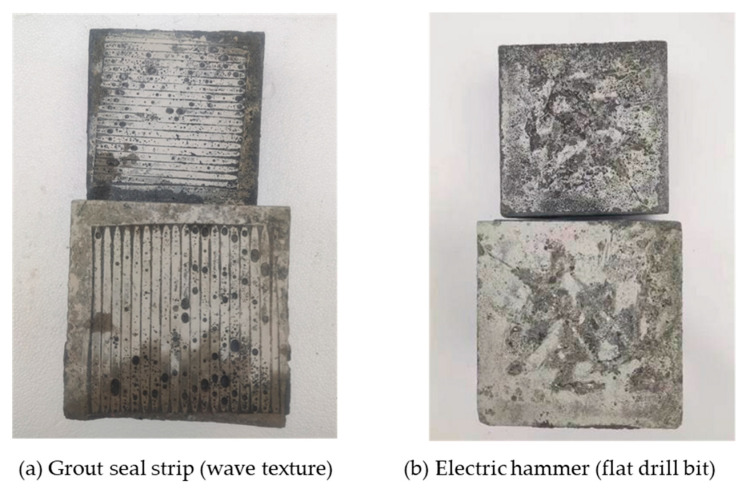
The surface characteristics of UHPC-NC shear specimens with mode A failure.

The mode A corresponds to the specimens treated by electric hammer (flat drill bit), water jet (after final set), grout seal strip (grass texture) and grout seal strip (wave texture), which have a small FTR. Typical specimens with mode A failure are shown in Figure 9. For these specimens, the roughening depth is relatively shallow and the interfacial bonding is relatively weak, which result in sliding during shear test. For this failure mode, the strength advantage of the UHPC was not effectively utilized.

**Figure 10:**
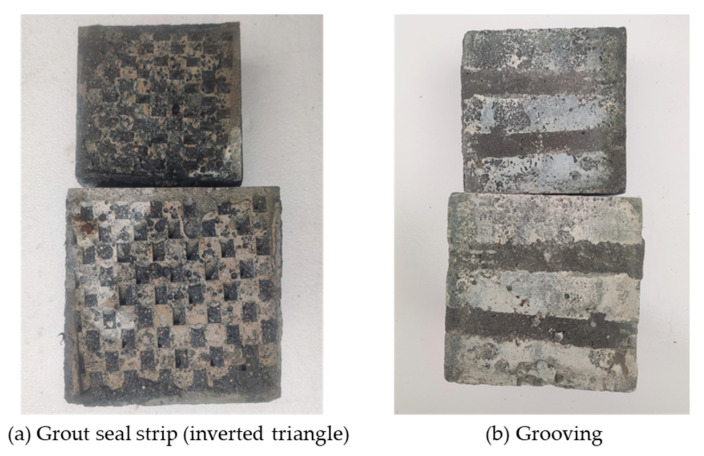
The surface characteristics of UHPC-NC shear specimens with mode B failure.

The failure mode B corresponds to the specimens treated by electric hammer (drilling), water jet (after final set), grooving and grout seal strip (inverted triangle). Typical specimens with mode B failure are shown in Figure 10. For these specimens, the roughening depth is deeper than that of mode A, however, the total area of groove is smaller than untreated area, so the NC has a size advantage in terms of shear capacity. As a result, the UHPC breaks at the interface and embeds in the NC.

**Figure 11:**
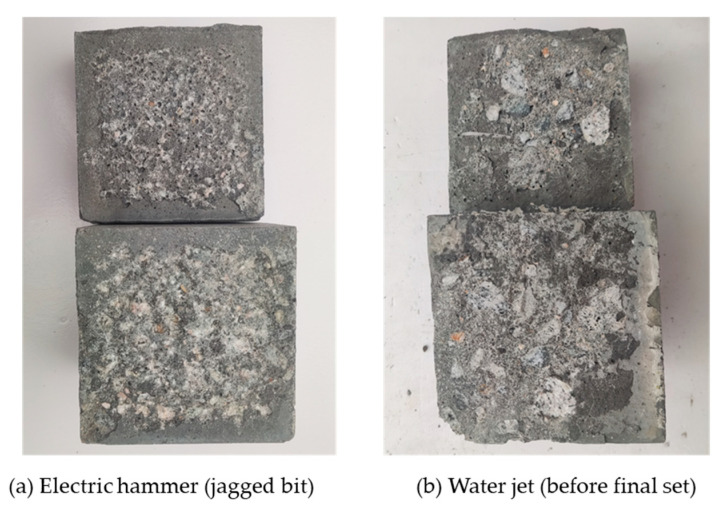
The surface characteristics of UHPC-NC shear specimens with mode C failure.

Mode C failure primarily occurred in specimens treated by electric hammer (jagged bit) and water jet (before final set). Typical specimens with mode C failure are shown in Figure 11. For the first condition, the electric hammer (jagged bit) may cause microcracks on NC surface, which leads to the spalling of NC. As for the second condition, the NC was treated by water jet before final set of NC, this may wash away the cement paste near aggregates and diminish the surface strength of the old concrete. Besides, the water jet also increases the porosity of the old concrete surface, these defects also diminish the shear capacity of old NC.

### 3.3. Relationship Between Shear Strength and Interface Roughness

**Figure 12:**
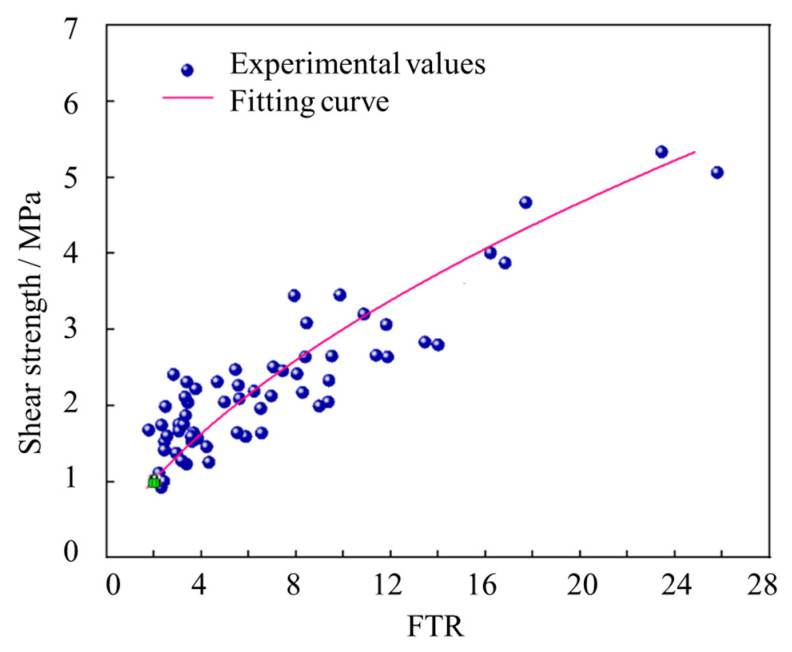
Fitting curve of roughness and shear strength for shear specimens.

In this work, a total of 61 NC-UHPC specimens were prepared and tested with our device, the roughness (indicated by FTR) and shear strength are listed in Figure 12. The experimental results were fitted with a quadratic polynomial using the least squares method, and the fitting function can be described as

(6) $$\tau =0.546FTR^{2}+1.832FTR-0.447,$$

The correlation coefficient is R^2^ = 0.831.

Based on the fitting results, it is evident that with the increase of FTR, the interfacial shear strength initially increases and then tends to moderate. This finding is consistent with the research findings of previous scholars [15]. Firstly, a higher surface roughness increased the bonding area between the new and old concrete, thereby providing greater shear resistance. Secondly, the uneven surface can play the role of a shear key, which also increase the interfacial shear strength. However, when the surface roughness reaches a certain level, the shear strength increase will gradually moderate due to the limitation of the inherent shear strength of the materials. In this work, the correlation coefficient of the fitting curve is R^2^ = 0.831, this indicates an undeniable error between experimental values and theoretical calculation values. Actually, due to the unevenness of concrete and other influencing factors, the experimental results exhibit significant variability. Despite this fact, the shear testing device offers good convenience in specimen preparation and testing operations and is sufficient to meet application needs.

## 4. Conclusions

This paper proposed a concrete shear testing device. The feasibility and reliability of the device are demonstrated via finite element simulation and the experimental test. Surface roughness is reported as one of the most important factors impacting the shear strength of new–old concrete. In order to quantify this relationship, old concrete blocks were treated using nine different methods to create different profiles and fresh concrete was cast atop to form the shear specimen. With the proposed device, the shear strength of new-to-old concrete was tested and the correlation between surface roughness and shear strength was quantified. From the findings of this paper, the following conclusions can be drawn:

(1) The finite element simulation results show that the increasing roughness improved the failure load and changed the stress distribution in new-to-old concrete. The improved failure load significantly increased the normal stresses of S22 and S33 since the specimen is subjected to both shear force and bending force which is caused by eccentric loading
(2) The failure mode of new–old concrete interface depends on the treatment method and roughness of the old concrete surface. The concrete specimens tend to fail at the interface when the roughness is small and the roughening depth is shallow. With the increase of surface roughness, the failure zone could occur on new or old concrete, depending on the of total area of groove and the strength of each material.
(3) The experimental results show that the shear strength at new–old concrete interface has a positive correlation with surface roughness and that the correlation between shear strength (τ) and Fourier transform roughness (FTR) can be described with the equation τ (MPa) = 0.546*FTR*^2^ + 1.832*FTR* − 0.447.
(4) The above results indicate that the shear testing device designed in this paper can be used for testing the shear performance of new–old concrete interfaces, offering good operability and reliable results. Additionally, this experimental method can provide some reference and guidance for shear experiments on other materials.

## Figures and Tables

**Table 1:** The compositions (kg/m^3^) and mechanical properties of the old concrete.

Concrete	Cement	Sand	Gravel	Water	Water Reducer	w/b	Strength (MPa)		
							7 d	28 d	60 d
C40	400	688	1076	156	9	0.39	34.2	45.2	54.6

**Table 2:** Parameter used in the CDP model.

Parameters	*ψ*	*ε*	*f_b_*_0_ */f_c_*_0_	*κ*	*μ*
Values	30	0.1	1.16	0.667	0.005

**Table 3:** Interface parameter table for different roughness levels.

	Smooth	Intermediate	Rough
*K_nn_* (N/mm^3^)	1372	2277	2559
*K_ss_* (N/mm^3^)	586	973	1093
*K_tt_* (N/mm^3^)	586	973	1093
*t_n_*^0^, *t_s_*^0^, *t_t_*^0^ (MPa)	3.02	5.01	5.63
Total displacement (mm)	0.018	0.117	0.241

**Table 4:** The maximum normal stresses at interfaces with different roughness levels.

	S11 (MPa)		S22 (MPa)		S33 (MPa)	
	Compression	Tension	Compression	Tension	Compression	Tension
Smooth	−0.14	0.63	−1.20	0.52	−0.27	0.86
Intermediate	−0.69	0.65	−3.58	0.53	−2.96	1.07
Rough	−1.01	0.61	−3.68	0.62	−2.74	1.25

**Table 5:** Average FTRs and failure modes of concrete treated by different methods.

Interface Treatment Methods	Average FTR	Failure Mode
Electric hammer (flat drill bit)	3.04	A
Electric hammer (drilling)	3.92	B
Electric hammer (jagged bit)	7.42	C
Water jet (before final set)	13.45	C
Water jet (after final set)	4.84	A,B
Grooving	11.32	B
Grout seal strip (inverted triangle)	11.37	B
Grout seal strip (grass texture)	3.85	A
Grout seal strip (wave texture)	2.87	A

## Data Availability

The raw data supporting the conclusions of this article will be made available by the authors on request, they are not publicly available due to privacy.

## Abbreviations

The following abbreviations are used in this manuscript:

PTFE	polytetrafluoroethylene
UHPC	ultra-high-performance concrete
FTR	Fourier transform roughness
NC	normal concrete
CDP	concrete damaged plasticity
